# A comparative study of cortico-cancellous iliac bone graft with or without the combination of vascularized greater trochanter flap for the management of femoral head osteonecrosis: a minimum 6 years follow-up

**DOI:** 10.1186/s12891-019-2613-1

**Published:** 2019-06-22

**Authors:** Wenjun Feng, Jinlun Chen, Keliang Wu, Lu Lu, Peng Deng, Pengcheng Ye, Houran Cao, Jie Li, Jianchun Zeng, Ke Jie, Xinyu Qi, Yirong Zeng

**Affiliations:** 1 Guangzhou University of Chinese Medicine, Jichang Road 16#, District Baiyun, Guangzhou, Guangdong China; 2 The First Affiliated Hospital of Guangzhou University of Chinese Medicine, Jichang Road 16#, District Baiyun, Guangzhou, Guangdong China; 3 Guangzhou University of Chinese Medicine, Jichang Road 12#, District Baiyun, Guangzhou, Guangdong China; 4 The First Affiliated Hospital of Guangzhou University of Chinese Medicine, Linnan Medical Research Center of Guangzhou University of Chinese Medicine, Jichang Road 16#, District Baiyun, Guangzhou, Guangdong China

**Keywords:** Osteonecrosis of the femoral head, Vascularized greater trochanter flap, Cortico-cancellous iliac bone graft, Hip-preserving, Outcomes comparison

## Abstract

**Background:**

To compare the mid-long-term clinical and radiological outcomes between a combination of cortico-cancellous iliac bone graft with vascularized greater trochanter flap (Group A) and isolate iliac bone graft (Group B) in the treatment of Osteonecrosis of the Femoral Head (ONFH).

**Methods:**

From January 2006 to December 2012, 123 patients (135 hips) who underwent abovementioned hip-preserving surgeries were included for analysis. Clinical outcomes were assessed based on Harris Hip Score (HHS) System and The Western Ontario and McMaster University Index (WOMAC) scores between the preoperative and the last follow-up. A series of postoperative X-rays were compared to preoperative images for radiological evaluation.

**Results:**

The HHS in Group A and B were enhanced from 50.57 ± 3.39 to 87.60 ± 4.15 and from 50.24 ± 3.30 to 85.18 ± 6.45, respectively, which both showed significance between preoperative and postoperative latest follow-up (*p* < 0.001). Group A revealed better improvement in terms of HHS (*p* = 0.017). The WOMAC total, postoperative stiffness, difficulty subscale scores in Group A showed better outcomes when compared to Group B (*p* < 0.01), while pain improvement between these two groups revealed no significance (*p* = 0.402). Besides, Group A suggested better necrotic region repair (*p* = 0.020), but no femoral head collapse difference in terms of Association Research Circulation Osseous classification change was found (*p* > 0.05).

**Conclusions:**

A combination of cortico-cancellous iliac bone graft and concurrent vascularized greater trochanter flap with the lateral femoral circumflex transverse branch has been proved can obtain better functional and radiological results than isolate iliac bone grafting, which is attributed to blood reconstruction of the femoral head.

## Background

Osteonecrosis of the femoral head (ONFH) is a common debilitating and disabling condition, which is characterized as blood supply impairment of the femoral head, bone marrow and osteocytes death, subchondral bone collapse, articular cartilage degeneration and ultimate secondary osteoarthritis [1, 2]. It mainly affects adults in their thirty and forty decades of life [3], with an estimated number over 20,000,000 worldwide today [4] and morbidity incidence ranged from 8.9/ 100,000 [5] to 28.9/100,000 [6]. Though alcohol intake [7], corticosteroid use [8], posttraumatic [9], bone marrow fat embolisms [10], hypercoagulation [11] and endothelial dysfunction [12] have been identified as its risk factors, the underlying etiology and pathogenesis is still not completely clear [13], which makes it difficult to predispose the development and advancement in gene and molecular level.

Once ONFH is confirmed, the intervention shall initiate immediately. Mont MA et al. [14] found that when treatment delayed, 70% cases will suffer progressive collapse in about 3 to 4 years according to the natural course of ONFH. Though Total Hip Arthroplasty (THA) has been proved to be an effective procedure in the treatment of ready collapse late stage ONFH [15, 16] with a mean 90% survivorship at 20 years in most implants [17], it poses great challenge on younger patients when considering prosthesis early loosen [18], revisions [19] and infections [20]^.^ Therefore, other hip-preserving surgeries aimed to delay and/or prevent THA conversion as long as possible in these young patients are needed. The goal of hip-preserving surgeries is to restore disordered bone metabolism and maintain stable structure support. Various surgeries have been proposed to improve necrosis bone repair and prevent or postpone progressive collapse, such as core decompression [21], proximal femur rotational osteotomy [22], vascularized [23] or non-vascularized [24] bone grafting, artificial biomaterials impaction [25], bone grafting combining with bone morphogenetic protein [26], and autologous bone marrow aspirate transplantation [27]. For these precollapse ONFH cases, bone grafting can not only provide mechanical support, but also improve necrotic region biological environment when necrotic tissues were thoroughly removed [28]. With regard to the bone grafting surgery, vascularized and non-vascularized options are available to perform mainly depended on surgeon preference. Alderdge et al. [29] discovered that revascularization of the necrotic region can stimulate osteoinductive progenitor cells invasion and promote bone revitalizing. Once blood supply is restored or reconstructed, vital bone can gradually creeping substitute the necrotic tissues and a relative normal subchondral plate is regained.

However, to our knowledge, no consensus has been reached concerning the superiority of mid-long-term efficacy in postcollapse ONFH cases between these two types of bone grafting techniques. Thus, we conducted a retrospective comparative study between cortico-cancellous iliac bone graft alone and in combination with concurrent vascularized greater trochanter flap based on the lateral femoral circumflex artery transverse branch, for the treatment of ONFH in Association Research Circulation Osseuse (ARCO) stage III at a minimum 6 years follow-up. As we have seen, no prior published studies have compared the clinical and radiological outcomes between these two surgeries. We asked the following questions: (1) What are the mid-long-term clinical and radiological outcomes of the abovementioned two techniques? (2) Which technique shows superiority in terms of improvement on Harris Hip Score (HSS), The Western Ontario and McMaster University Index (WOMAC) total and subscale scores, extent of femoral head collapse and necrotic region repairment? (3) What is the survivorship of each technique when consider THA conversion as the endpoints?

## Methods

Patients who underwent hip-preserving surgeries were retrospectively reviewed at the First Affiliated Hospital of Guangzhou University of Traditional Chinese Medicine from January 2006 to December 2012. This study was approved by the Ethics Committee of Guangzhou University of Traditional Chinese Medicine and it complied with the Declaration of Helsinki. Patients who presented in ARCO stage III with age between 18 years and 45 years and underwent previous described two surgical procedures were considered as the inclusion criteria. Candidates who underwent a combination of iliac bone and vascularized greater trochanter flap with lateral femoral circumflex artery transverse branch and isolate cortico-cancellous iliac bone graft and were divided into Group A and Group B, respectively. The exclusion criteria consisted of previous hip invasion surgeries (such as core decompression, stem cell injection, endovascular intervention), tumor, metabolic bone disease, hip infections, history of blood vessel structure and/or function disorder, unable to follow postoperative training programs and incapable of understanding instructions due to mental health deficiency.

One hundred and twenty seven patients (139 hips) were recruited in the current study. In Group A, 1 patients (1 hip) died after postoperative 10 months attributed to unrelated surgical cause, and 1 patient (1 hip) was eliminated for further analysis because of corticosteroids pulse therapy due to primary disease relapse. There was one patient (1 hip) in each group was lost follow-up. Therefore, 123 patients (135 hips, 97.1%) were enrolled for finally assessment. There were 51 men (55 hips) and 72 women (80 hips), and patients completed regular instructed revisit in the out-patient department. The demographics data consisted of number of patients (hips), age (at the time of surgery), sex, Body Mass Index (BMI), affected sides, etiologies in both groups were presented in Table 1.

**Table 1:** Comparison of baseline characteristics of Group A and B

Characteristics	Group A	Group B	*P* value
No. of patients(Hips)	77 (84)	46 (51)	
Age (years; mean ± SD, [range])	33.2 ± 5.7 (18–45)	32.8 ± 5.0 (18–45)	0.724
Sex(female/male)	46/31	26/20	0.850
BMI(kg/m^2^)	22.0 ± 1.2 (19.5–25.4)	22.3 ± 1.3 (19.8–24.9)	0.335
F-U time (years; mean ± SD, [range])	9.7 ± 1.4 (6–12)	9.8 ± 1.3 (7–12)	0.789
Affected hips (Right/Left)^a^	48/36	27/24	0.722
Risk factors			
Alcohol	24	15	> 0.999
Corticosteroid	36	20	0.852
Idiopathic	17	11	0.827
ARCO stage			
IIIA	29	18	0.537
IIIB	41	21	
IIIC	14	12	

*BMI* Body Mass Index, *F-U* Follow-Up, *ARCO* Association Research Circulation Osseous. ^a^7 patients had bilateral involved hips in Group A, while Group B had 5 patients with bilateral affected hips. *P* < 0.05 indicates significance

### Surgical procedure

All surgeries were performed by one same senior surgeon. The complete procedure has been described in previous study [30] and was categorized as 3steps. In briefly, in the first step, a modified Smith-Peterson approach was used for exposure. The lateral femoral circumflex artery transverse branch was identified and separated from surrounded soft tissues carefully, followed by the greater trochanter flap was chiseled with arc-shaped osteotome along the pedicle parts. In the second step, an opening window-like approach access to the necrotic region was obtained. The necrotic tissues were thoroughly removed and the hardening boundary was destructed via a 1.5-mm Kirschner wire drilling. In the third step, a rectangular cortico-cancellous iliac bone graft was harvested according to the necrotic size and was cut into half to fit the necrotic space. The remaining gap was filled with allogeneic cancellous bone tightly impaction grafting and the prepared vascularzied greater trochanter flap was implanted. The femoral head was shaped more congruent and reduced. For patients in Group B, the surgical procedure was the same as Group A except for the vascularized greater trochanter flap preparation and implantation. Patients were not allowed weight-bearing at the first 6 weeks followed by partial weight-bearing till the 6th postoperative month. Full weight-bearing was allowed after the 6th postoperative month.

### Outcome evaluation

Outcome evaluation was performed by two independent investigators. All patients follow-up was scheduled at 3, 6, 12 months and yearly thereafter. HHS and WOMAC (pain, stiffness and difficulty subscales) scores was used for clinical evaluation. A series of anteroposterior and frog lateral X-rays of bilateral hips at preoperative and each follow-up were used for radiographic evaluation. Femoral head morphology and necrotic region repair process were the primary objectives of radiological evaluation. We defined reduced or disappeared necrotic cystic lesions without osteoarthritis (OA) as improved, and reduced or stabled necrotic cystic region with no or slight OA as stabilized, and enlarged necrotic region with apparent OA as aggravated. It was defined as clinical failures of these two surgeries when subsequent THA was indicated. Besides, the complications were also recorded.

### Statistical analysis

All statistical analysis was performed using SPSS Statistical software (Version 18.0, IBM Cooperation, USA). Distributions of quantitative variables were expressed as means (±SD) or by median and interquartile range. For intra- group analysis, the Wilcoxon Test was used to compare the preoperative and the last postoperative HSS and WOMAC scores in these two groups, while the Mann-Whitney U Test was used for inter-groups preoperative baseline and postoperative difference analysis. Qualitative variables were summarized as count and percentage and compared with Fisher’s exact test. A *P*-value less than 0.05 were considered to be statistical significance.

## Results

The preoperative and the latest follow-up HHS, WOMAC total scores and subscale scores in both groups were presented in Table 2. The HHS in both groups revealed significant statistical difference when compared to preoperative levels (*p* < 0.001). Besides, Group A revealed more rate of improvement than Group B in the aspect of HHS difference (Δ HHS equal to the latest follow-up HHS minus preoperative scores, *p* = 0.017). The last postoperative WOMAC total and subscale scores (pain, stiffness and difficulty) showed significant improvements when compared to the preoperative baseline in both groups (*p* values were all less than 0.001). Group A showed more superior results than Group B in terms of total, subgroup stiffness and difficulty enhancement (*p* < 0.01), while the difference of WOMAC subscale pain scores between these two groups revealed no significance (*p* = 0.402).

**Table 2:** HHS, WOMAC total and subscale scores in both groups

Parameters	Group A				Group B				*p* value for intergroup
	Mean ± SD			*p* value for intragroup	Mean ± SD			*p* value for intragroup	
Pre-HHS	50.57	±	3.38	**< 0.001** ^*^	50.24	±	3.30	**< 0.001** ^*^	0.017^*^
Post-HHS	87.60	±	4.15		85.18	±	6.45		
Pre-WOMAC	90.52	±	8.22	**< 0.001** ^*^	87.00	±	7.98	**< 0.001** ^*^	**< 0.001** ^*^
Post-WOMAC	36.75	±	6.70		39.90	±	5.55		
Pre-pain	11.68	±	2.37	**< 0.001** ^*^	11.33	±	2.73	**< 0.001** ^*^	0.402
Post-pain	1.96	±	1.28		1.88	±	1.29		
Pre-stiffness	11.17	±	2.16	**< 0.001** ^*^	12.63	±	1.44	**< 0.001** ^*^	**< 0.001** ^*^
Post-stiffness	2.99	±	2.16		2.90	±	1.32		
Pre-difficulty	68.12	±	5.05	**< 0.001** ^*^	68.53	±	4.30	**< 0.001** ^*^	0.008^*^
Post-difficulty	32.63	±	4.14		35.29	±	3.00		

*HHS* Harris Hip Score, *WOMAC* Western Ontario and McMaster University Index. Wilcoxon test was used for preoperative and the last postoperative intra-groups comparison, while Mann-Whitney U test was used for intergroup and delta difference comparison

**Fig. 1:**
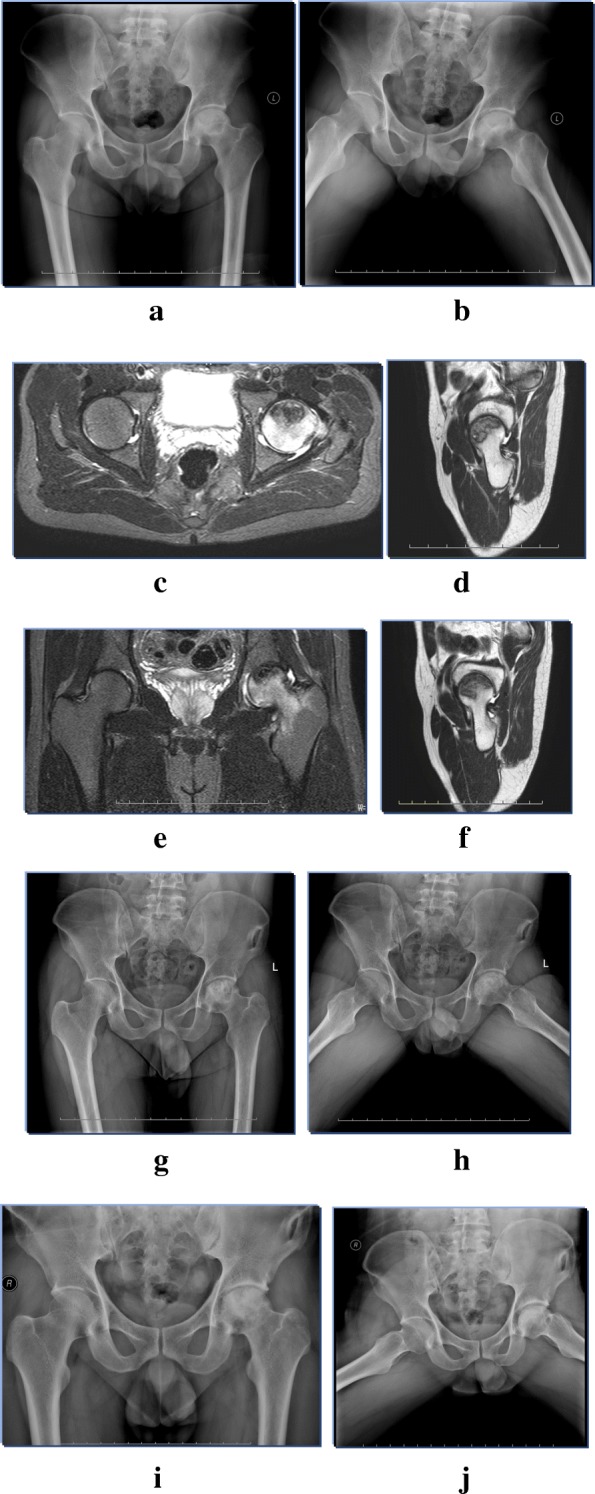
(**a**, **b**) Anteroposterior and frog lateral X-rays of bilateral hips showed that left hips were in ARCO stage IIIC. (**c**, **d**, **e**, **f**) preoperative MRI images showed left hip was involved serious ONFH. (**g**, **h**) the immediate radiographs followed by free iliac flap grafting combining with vascularized greater trochanter implantation of left hip were shown. (**i**, **j**) intact joint space was achieved with matched femoral head morphology at10 years postoperatively, and the left hip was successfully preserved

**Fig. 2:**
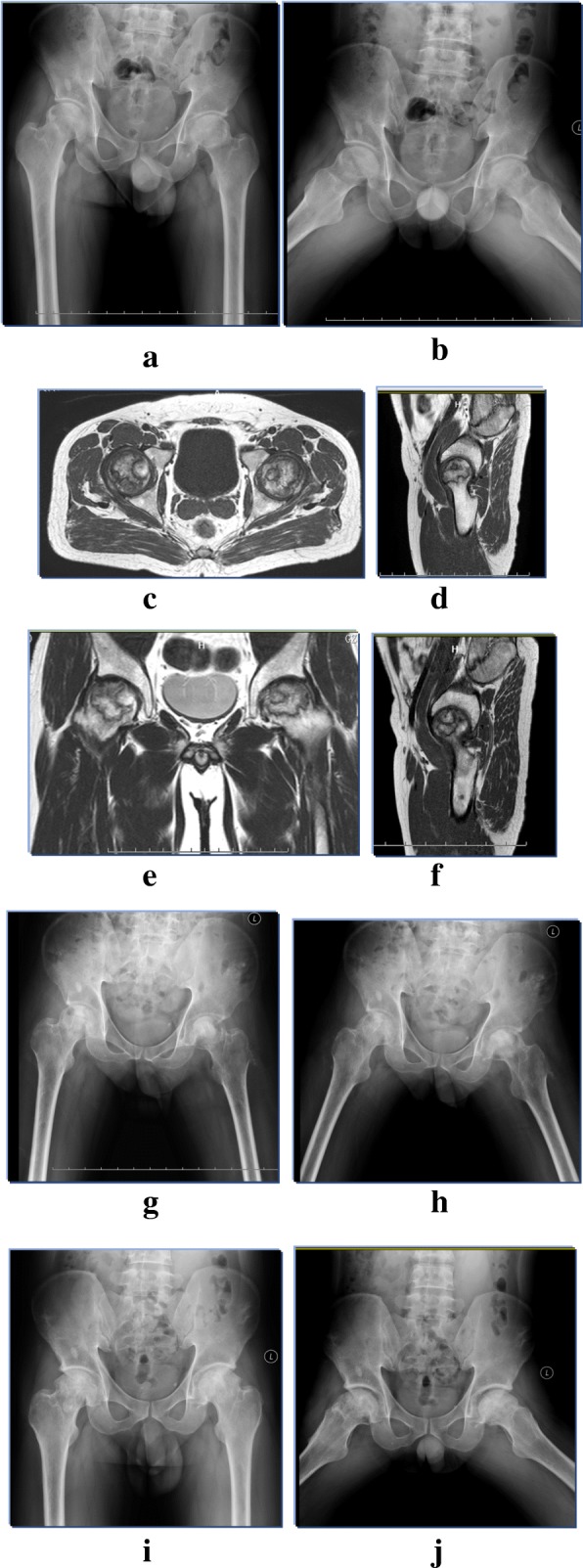
(**a**, **b**) anteroposterior and frog lateral X-rays of bilateral hips showed that both hips were involved with ONFH. 2 (**c**, **d**, **e**, **f**) preoperative MRI revealed that both involved hips were in ARCO stage IIIC. (**g**, **h**) the immediate radiographs after isolate free iliac bone grafting were presented. (**i**, **j**) the congruent femoral head shape was presented without secondary osteoarthritis at 7 years postoperatively

ARCO classification system was used for evaluation of the extent of femoral head collapse, which was summarized in Table 3. In terms of femoral head collapse, both groups showed significant improvement in the latest postoperative follow-up when compared to preoperative femoral head shape (*p* = 0.046 and 0.040, respectively). However, no significance was presented between these two groups from the aspects of femoral head collapse (*n* = 27 and 14, respectively, *p* > 0.05). The improved, stabilized and aggravated cases in Group A were 19, 38, 27. While in Group B, 23 cases were improved, 14 cases were stabilized, and 14 cases were aggravated (Fig. 1 and Fig. 2). Group A revealed better repair capability (*p* = 0.020, Table 4).

**Table 3:** Comparison of radiological outcomes in terms of the extent of femoral head collapse between preoperative and the last postoperative follow-up in Group A and B

ARCO classification	Group A		*P* value	Group B		*P* value	*P* value for improvement a
	preoperative	postoperative		preoperative	postoperative		
IIIA	29	45	0.046	18	31	0.040	0.568
IIIB	41	30		21	14		
IIIC	14	9		12	6		

*ARCO* Association Research Circulation Osseous. a The comparison between Group A and B in terms of ARCO classification improvement was analyzed by Chi-square test. In Group A, 4 hips in ARCO IIIA were aggravated, 17 hips was stabilized, 8 hips were improved. 13 hips in ARCO IIIB were aggravated, 19 hips was stabilized, 9 hips were improved. 10 hips in ARCO IIIC were aggravated, 2 hips was stabilized, 2 hips were improved. In Group B, 3 hips in ARCO IIIA were aggravated, 7 hips was stabilized, 8 hips were improved. 5 hips in ARCO IIIB were aggravated, 4 hips was stabilized, 12 hips were improved. 6 hips in ARCO IIIC were aggravated, 3 hips was stabilized, 3 hips were improved

**Table 4:** Comparison of radiological outcomes from the aspect of necrotic region repair between Group A and B

Postoperative necrotic region repair	Group A	Group B	*P*
Improved	19	23	0.020
Stabilized	38	14	
Aggravated	27	14	

THA conversion was defined as failure of these two surgeries. 6 hips (7.14%, 6/84)in Group A accepted THA at a mean follow-up of 6.8 years (range, 6–8 years). Among the conversion hips, 3 hip was in ARCO stage IIIB and 3 hips were in ARCO stage IIIC at the time of surgery. There were 11 hips (21.6%, 11/51) had THA conversion Group B with an average follow-up of 6.5 years (range, 6–8 years). 1 of these hips had ARCO stage IIIA, 4 hips had ARCO stage IIIB, and the other 6 hips had ARCO stage IIIC. Group A showed better survivorship than Group B (*P* = 0.030).

One superficial wound infection occurred in Group A and healed after extended antibiotics administration. 2 patients complained prolonged greater trochanter donor pain, which finally complete relieved through physical therapy and oral analgesics administration. No greater trochanter or pelvic fracture occurred. Each group had 2 patients with numbness and hypoesthesia in the lateral parts of thigh, which resolved with time.

## Discussion

ONFH is a commonly seen intractable condition, which mainly affects young and middle-aged individuals and makes the treatment very challenging. Though THA has been known as one of the most successful and creative techniques in twentieth century, it does put young patients into great risks of implant early loosen, infections and revisions [18–20]. The primary purpose of hip-preserving surgeries is to delay and/or prevent THA conversion. Various hip-preserving surgical options have been proposed, however, the most-effective treatment is still under great controversies [31].

The principle of hip-preserving surgeries for prevention of femoral head collapse and promotion of necrotic region repairment consisted of complete necrotic bone removal, trabecular bone reconstruction, hip biomechanics restoration as well as femoral head blood supply recovery [32]. Necrotic thoroughly debridement combining with bone grafting shows the potential of preventing femoral head collapse through reconstructs mechanical support. In 1930, Phemister was the first surgeon that using non-vascularized fibular bone grafting in the treatment of ONFH, which based on the theory that the implanted bone would not only provided mechanical support, but also could be the scaffold for progenitor cells adhesion and new bone creeping substitution [33]. Many new techniques had been documented since then. Trousdale RT [34] found that vascularized bone grafting showed more superior results than non-vascularized bone grafting, which was further confirmed by Kim [35], Mastusaki H [36], Hasegawa Y [37] and so on. The underlying theory might attribute that blood supply reconstruction would improve circulating stem cells and growth factors delivery, which would increase necrotic region new vital bone formation and promote implanted bone healing [38]. Since the advantages of vascularized bone grafting were introduced, numerous of new options have been described, such as the vascularized greater trochanteric flap, free vascularized fibular graft, iliac crest vascularized graft. Though vascularized bone grafting of all types have been reported to be effective, results from each center were varying. Consequently, new modifications of these techniques are needed.

The vascularized greater trochanter flap grafting is not commonly employed among the abovementioned options [30, 39], it did significant improve HHS and SF-36 scores in postcollapse ONFH patients, and the THA conversion rate at a mean 8 years follow-up was 11.8% (23/195). Zhao D et al. [40] conducted an anatomic study and found that the outer diameter of lateral femoral circumflex artery transverse branch was 2.5 ± 0.8 mm with a sufficient length of 3.5 ± 0.8 mm, which made it less damage during surgery. The vascularized greater trochanter owned reliable and abundant blood supply. He used the method to treat 32 ONFH patients in Ficat stage II-III, discovered that the clinical success rate was 90.6% and radiographic success rate was 87.5%. To us known, a comparative study to detect the efficacy between isolate free iliac bone flap with or without concurrent vascularized greater trochanter grafting with femoral circumflex transverse branch has not been documented so far.

In our study, we found that patients obtained very good improvement of HSS at an average 9.7 years follow-up in both Group A and B, which suggested that both techniques showed efficacy in the treatment of ONFH. The good-excellent rate (defined as HHS ≥80) in Group A and B was 87.0% (73/87) and 70.6% (36/51), respectively. The Group A showed better HHS improvements, which might be attributed to more efficient repair response due to vascularized greater trochanter flap implantation, though more deep evident studies were needed. In terms of Patient-Reported Outcomes (PROMs) evaluation, all patients in both groups achieved pain relief. However, patients in Group A obtained more improvements in the aspects of and stiffness and daily quality difficulty. We concluded that the implantation of the vital lateral femoral circumflex transverse branch played a crucial role in the necrotic bone repair, implanted bone revascularization and biomechanical stability restoration, especially in the first postoperative year. Though both groups had significant case number change of ARCO subtypes, Group A showed no superior radiological performance than Group B, which might be accounted to small samples in both groups. Further studies concerning digital subtraction angiography, PECT/CT and Magnetic Resonance angiography might provide more firmed evidence regarding the vascularity of implanted lateral femoral circumflex artery transverse branch [41]. The postoperative survivorship at an average 9.7 years follow-up between these two groups did not have significance (92.8% vs 78.4%), but both groups showed comparable hip-preserving success. We considered that complete necrotic bone removal, free iliac bone flap sufficient support, allogeneic cancellous bone tight impaction grafting and vital blood circulation of lateral femoral circumflex artery transverse branch pedicled with greater trochanter as the key point to obtain long-term success. 2 patients complained persistent pain in Group A, which, in our opinion, might be attributed to the decrease of resistance to tensile stress oriented from surrounding muscles due the greater trochanter integrity destruction.

The current study has several limitations. First, it was a case-cohort retrospective comparative study. The case number of both groups is relatively small. Though the mean follow-up time in both groups is near 9.7 years, the number of follow-up time beyond 10 years in the study and control group was 25 hips (29.8%, 25/84) and 12 hips (23.5%, 12/51), respectively. Our study showed that both hip-preserving techniques can obtain good efficacy and prevent or delay THA conversion. However, we need a randomization study, larger patient samples and longer follow-up time to reduce the bias and validate the outcomes. Second, a lack of postoperative computerized tomographic (CT) scans and MRI evaluation, which can assess the bony microstructure change and cartilage condition. Although a series of postoperative X-rays can provide enough message regarding the necrotic region repairment, extent of femoral head collapse, hip joint space and osteoarthritis appearance, a more comprehensive imaging evaluation might help to make a better objective estimation, especially for cartilage status assessment. Third, we did not assess the relationship between the size and location of necrotic lesion and postoperative clinical and radiological outcomes. Univariate and multivariate liner correlation studies might be beneficial to distinguish risk factors. Regardless of the limitations, we recommended the combination of cortico-cancellous iliac bone graft and vascularized greater trochanter flap grafting with the lateral femoral circumflex artery transverse branch in the treatment of ONFH in ARCO stage III. It is extremely important to make a systematic review of current available techniques to conclude a consensus of indications, contradictions, proper surgical interventions in different ARCO stages for purpose of better outcomes and more consistent results.

## Conclusions

For ONFH in ARCO stage III, both hip-preserving techniques showed good results in terms of hip function preservation. However, a combination of cortico-cancellous iliac bone graft and concurrent vascularized greater trochanter flap with the lateral femoral circumflex transverse branch has been proved can obtain better functional and radiological results.

## Abbreviations

ARCO: Association Research Circulation Osseous
BMI: Body Mass Index
HHS: Harris Hip Score
ONFH: Osteonecrosis of the Femoral Head
PROMs: Patient-Reported Outcomes
THA: Total Hip Arthroplasty
WOMAC: The Western Ontario and McMaster University Index
